# Temporal single-cell RNA sequencing dataset of gastroesophagus development from embryonic to post-natal stages

**DOI:** 10.1038/s41597-024-04081-7

**Published:** 2024-11-16

**Authors:** Pon Ganish Prakash, Naveen Kumar, Rajendra Kumar Gurumurthy, Cindrilla Chumduri

**Affiliations:** 1 Laboratory of Infections, Carcinogenesis and Regeneration, Medical Biotechnology Section, Department of Biological and Chemical Engineering, Aarhus University, Aarhus, Denmark; 2 Department of Microbiology, University of Würzburg, Würzburg, Germany

**Keywords:** Developmental biology, Data processing

## Abstract

Gastroesophageal disorders and cancers impose a significant global burden. Particularly, the prevalence of esophageal adenocarcinoma (EAC) has increased dramatically in recent years. Barrett’s esophagus, a precursor of EAC, features a unique tissue adaptation at the gastroesophageal squamo-columnar junction (GE-SCJ), where the esophagus meets the stomach. Investigating the evolution of GE-SCJ and understanding dysregulation in its homeostasis are crucial for elucidating cancer pathogenesis. Here, we present the technical quality of the comprehensive single-cell RNA sequencing (scRNA-seq) dataset from mice that captures the transcriptional dynamics during the development of the esophagus, stomach and the GE-SCJ at embryonic, neonatal and adult stages. Through integration with external scRNA-seq datasets and validations using organoid and animal models, we demonstrate the dataset’s consistency in identified cell types and transcriptional profiles. This dataset will be a valuable resource for studying developmental patterns and associated signaling networks in the tissue microenvironment. By offering insights into cellular programs during homeostasis, it facilitates the identification of changes leading to conditions like metaplasia and cancer, crucial for developing effective intervention strategies.

## Background & Summary

The gastroesophageal squamo-columnar junction (GE-SCJ) is a pivotal anatomical site where the stratified squamous epithelium of the esophagus meets the glandular columnar epithelium of the stomach. Functioning as a crucial anti-reflux barrier, it prevents the backward flow of acidic stomach contents into the esophagus^1^. Disruptions in its homeostasis, as observed in gastroesophageal reflux disease (GERD), can compromise this barrier, leading to mucosal damage^2,3^. In response to such prolonged damage, the esophageal epithelium may undergo metaplasia, giving rise to Barrett’s esophagus (BE), a condition where the native stratified squamous epithelium of the esophagus is replaced by non-native columnar cells resembling those found in the stomach or intestine^4^. BE often manifests near the GE-SCJ and is a precursor to esophageal adenocarcinomas^5,6^.

Understanding the histogenesis, maintenance, and regeneration of the two distinct epithelial lineages at the squamo-columnar epithelial borders of GE-SCJ, as well as the role of the underlying stroma, is crucial to decipher pathogenesis mechanisms at these sites^7^. Complex interactions among the various cell types within the underlying ecosystem regulates the evolution and maintenance of the GE-SCJ and during tissue injury or repair^8–10^. Any alterations in these interactions play a significant role in pathological disorders like BE and upper gastrointestinal tract carcinomas. The emergence of single-cell RNA sequencing (scRNA-seq) technology has advanced our understanding of such signalling events and the cellular dynamics within local microenvironments, providing invaluable insights^11,12^. Recent scRNA-seq studies on BE and upper gastrointestinal tract carcinomas predominantly focus on the epithelial tissue alterations^13–16^. However, the heterogeneity of fibroblasts and their role in defining epithelial homeostasis during healthy GE-SCJ development remain largely unexplored. To identify the driving mechanisms of pathological disorders such as BE and cancers, it is crucial to first understand the complex networks of cell-cell interactions and regulatory principles governing the evolution and maintenance of the healthy GE-SCJ.

**Fig. 1:**
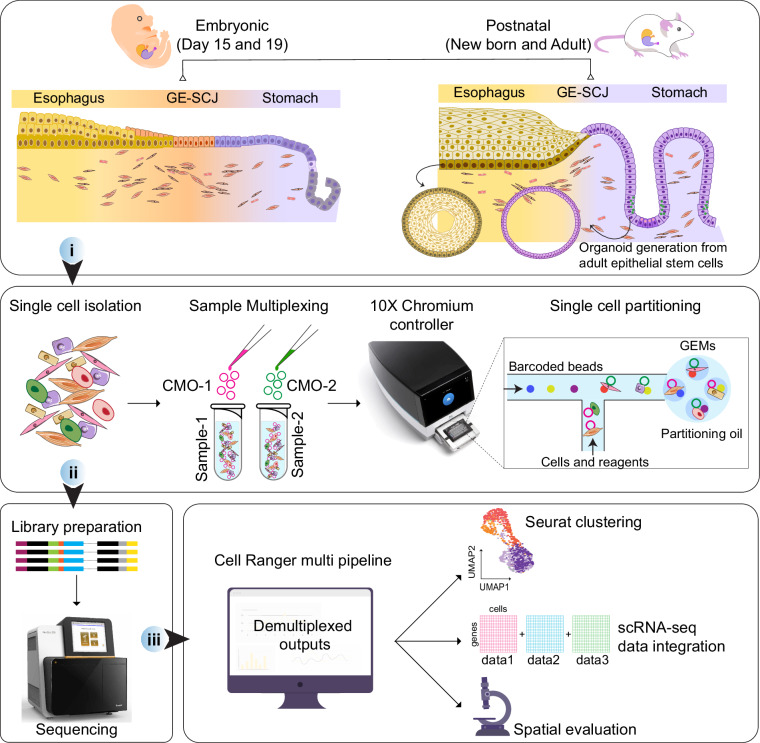
Schematic overview of experimental design. Graphics depict the esophagus, stomach, and the GE-SCJ tissue regions from mice at distinct developmental stages, including gestational days 15 and 19, newborn (2 days old), and adult (8 weeks old). Further, esophagus and stomach organoids were derived from adult epithelial stem cells to validate and complement physiological relevance. (i). single-cell preparation and capture: The initial step involves tissue or organoid digestion to obtain single cells and subsequent multiplexing of samples using the CMO (cell multiplexing oligos) technique followed by single-cell capture and barcoding using the 10X Genomics Chromium controller. (ii). Library preparation and sequencing: High-quality libraries for 3′ scRNA-seq are prepared and subjected to sequencing. (iii). Data processing and analysis: Upon sequencing, reads are aligned to the reference genome, and samples are demultiplexed, yielding a gene-by-cell count matrix. This matrix is then utilized in the Seurat workflow, facilitating robust data integration and spatial evaluation to measure the consistency and reliability of observations across datasets.

To bridge this knowledge gap, we conducted experiments using mouse models at various developmental stages, including embryonic day 15, 19, newborn (Pup), and adult, isolating tissues from the gastroesophageal region (Fig. 1). Employing 10X Genomics sample-multiplexed scRNA-seq technique, we generated extensive time-series data of the three tissue regions: the esophagus, stomach, and GE-SCJ^17^. Additionally, we utilized cutting-edge 3D esophagus and stomach organoid models derived from adult epithelial stem cells of mice. These mini-organ models faithfully replicate *in vivo* epithelial tissue architecture, providing invaluable insights complementing the data derived from respective native tissues. Consequently, the generated scRNA-seq data from esophageal and stomach organoids will also serve as a valuable data resource for further studies to elucidate the epithelial lineages, cellular heterogeneity, and their transcriptional characteristics. Our recent study^17^, utilized these datasets to investigate the evolution of epithelial and fibroblast subtypes, their transcriptional landscapes, cellular differentiation processes, and interactions between epithelial and fibroblast cells during healthy GE-SCJ development. This rich dataset, derived from all three regions of the gastroesophageal junction in both embryonic and adult stages, not only enhances our comprehension of the cellular dynamics and transcriptional signatures of a healthy GE-SCJ but also serves as an invaluable resource for future studies for unraveling the complex network of cell-cell morphogenetic signaling and the regulatory factors involved in its development from embryonic to adult stages and disease development.

In this Data Descriptor, we present insights into the technical quality of this time-series scRNA-seq dataset covering three distinct regions of the GE-SCJ. It emphasizes library quality, robustness, compatibility with other publicly available scRNA seq datasets, and their reusability, providing a more comprehensive resource for researchers in the field. We provide a detailed assessment of sample-specific sequencing quality metrics, including Q30 scores, read mapping percentages, and cell quality parameters. By performing integrative analyses of epithelial and stromal cells from both our dataset and external scRNA-seq sources, we demonstrate the dataset’s compatibility, underscoring its potential for cross-comparative studies. Further, we highlight the dataset’s applicability for studying developmental progression through lineage inference analysis, indicating its capacity to capture subtle developmental stage differences alongside cell-cell transcriptional variations. By performing cell types and marker gene expression validation using immunostaining experiments, we further confirmed the preservation of biological information during integration. This dataset also holds information on temporal gene expression profiles and cell-cell communication across various cell types, serving as a valuable resource for validating computational models of tissue histogenesis and curating cell-cell communication databases. Additionally, it provides opportunities for identifying changes and mechanisms related to tissue injury from gastric reflux and subsequent repair, as well as in infections and cancer development. Understanding these processes will facilitate identifying potential targets or biomarkers for early intervention and treatment of gastroesophageal diseases and pre-neoplastic events.

## Methods

Methods for epithelial cell and stomach gland isolation, organoid culture, immunofluorescence, smRNA-ISH, microscopy, tissue collection, single-cell preparation, and sequencing were as previously published^17,18^.

### scRNA-seq data processing and analysis of GE-SCJ tissue

The raw data processing of scRNA-seq data obtained from 10X Genomics was conducted using Cellranger (v.6.1.1) software. The standard pipeline ‘cellranger multi’ with default parameters was used for alignment, filtering, sample demultiplexing, barcode counting, and unique molecular identifier (UMI) counting of the single-cell FASTQ files. The mm10-2020-A genome build was used as the reference during read alignment. Following the generation of the UMI count matrix, quality control (QC), dimensionality reduction, and cluster analysis were performed using the R package Seurat (v.4.1.1)^19^. Initially, cell quality was assessed by performing correlation analysis between the number of genes and mitochondrial content per cell within each sample using the ‘FeatureScatter’ function. Individual levels of UMI counts, gene counts, and mitochondrial reads in cells across each sample were visualized using the ‘VlnPlot’ function. Based on these analyses, the scRNA-seq data was subjected to filtering to eliminate poor-quality cells or doublets, defined as cells with <100 genes, >8500 genes, or >80,000 UMIs. Cells exhibiting a mitochondrial gene percentage exceeding 20% were also excluded. This resulted in 7,808 total cells. Normalization and scaling were conducted using the sctransform (v.0.3.3) approach^20^, as detailed by the standard Seurat workflow, which involved regressing out the effects of cell cycle and mitochondrial reads. For pseudotime analysis, we modeled developmental trajectories using the default pipeline provided by the R package Slingshot (v.1.6.1)^21^, focusing on fibroblast cells from the esophagus tissue from different developmental time points. The starting point for this analysis was defined as cells from the E15 time point using the ‘start.clus’ parameter.

### scRNA-seq data integration

Publicly available scRNA-seq datasets from external research studies were downloaded for integrative analysis with our generated data. Specifically, we used the dataset for adult stomach stromal cells from the Gene Expression Omnibus (GEO) repository under accession code GSE116514^22^, and the dataset for adult stomach epithelia under accession code GSE157694^23^. The esophageal pup epithelia dataset was accessed from the ArrayExpress repository under the accession code E-MTAB-8662^24^, and the adult esophageal stromal cells dataset was retrieved from the Genome Sequence Archive (GSA, http://gsa.big.ac.cn) under accession code CRA002118^25^. Integration of these external datasets with our scRNA-seq data was achieved using the standard Seurat SCT integration workflow. The rPCA-based integration approach was used, utilizing the ‘PrepSCTIntegration’, ‘FindIntegrationAnchors’, and ‘IntegrateData’ functions of Seurat. Prior to integration, each dataset underwent individual normalization using the SCT approach, and low-quality cells were removed as previously specified. To mitigate biases arising from cell type proportion disparities across datasets, 10% of cells from each cell cluster were downsampled from all external data sources (except for stomach epithelia^23^, where 50% cells were downsampled), ensuring retention of all cell types and their respective gene signatures. Post-integration, we extracted epithelial and stromal cell clusters and performed combined re-clustering analysis using the same approach. Epithelial cell clusters were identified by the expression of markers such as *Trp63*, *Krt5*, *Krt13*, *Krt8*, and *Krt18*, while the stromal cell cluster was identified using the expression of *Dcn*, *Vim*, and *Acta2*.

Cell type annotations for all identified clusters was performed based on canonical marker gene expression or co-expression, supplemented by information from the Human Protein Atlas database. Further, the clustered gene expression plots were generated using the R package scCustomize^26^.

### scRNA-seq data of gastroesophageal organoids

For scRNA-seq data generated from mouse esophagus and stomach organoids, a similar processing and analysis pipeline was employed with slight modifications. Raw data processing was conducted using the 10X Genomics software CellRanger (v3.1.0), utilizing the built-in commands “cellranger mkfastq” and “cellranger count” to generate the feature-barcode matrix. The reference genome assembly used for read alignment was mm10-2020-A. In contrast to the tissue scRNA-seq data, where we utilized the 10x Genomics 3′ CellPlex Kit for sample multiplexing, we employed the lipid-tagged indices of the MULTI-seq technique^27^ for multiplexing organoid samples. Consequently, demultiplexing was carried out using the R package demultiplex (v1.0.2) (https://github.com/chris-mcginnis-ucsf/MULTI-seq) with default settings. Individual feature-barcode matrices were imported into the R environment using the ‘Read10X’ function, followed by the creation of Seurat objects for each sample. Similar to the tissue scRNA-seq data processing, cell quality in the organoids was assessed through correlation analysis between the number of genes and mitochondrial content within each sample using the ‘FeatureScatter’ function. UMI counts, gene counts, and mitochondrial reads per cell across samples were visualized using the ‘VlnPlot’ function. QC was performed using the same filtering criteria detailed above, leading to a total of 855 cells. Normalization was executed using Seurat (v4.0.0)^19^, following the ‘SCTransform’ approach. Dimensionality reduction and clustering were performed using the ‘RunPCA’, ‘FindNeighbors’, and ‘FindClusters’ functions with default parameters on the top 30 principal components. The non-linear dimensionality reduction function ‘RunUMAP’ was then applied to visualize the data.

## Data Records

### 10X Genomics scRNA-seq data of GE-SCJ tissue

The feature-barcode raw UMI count matrix from 10X Genomics scRNA-seq experiments of the GE-SCJ tissues isolated from E15, E19, pup, and adult mice is available in the GEO repository under the accession code GSE227412^28^. All files associated with both gene expression and CMO libraries are compressed in a gzip archive format with the filename ‘GSE227412_RAW.tar’. Within this archive, the raw count matrix is provided in the MTX format, consisting of the files ‘GSM7100143_barcodes.tsv.gz’,’GSM7100143_features.tsv.gz’and’GSM7100143_matrix.mtx.gz’. Furthermore, the corresponding sample demultiplexed data, including cell-sample-CMO summary, sample-barcode count information, and associated metadata, are available in CSV format. These files are named: ‘GSM7100144_Feature_Reference_GEJ_Tissue.csv.gz’, ‘GSM7100144_GEJ_CMO_SampleID_Summary.csv.gz’,’GSM7100144_Sample_Cell_Barcode_Assignment.csv.gz’ and ‘GSM7100144_GEJ_Seurat_Metadata.csv.gz’, respectively. These files can be accessed using any text editor or spreadsheet software. Upon retrieval, the count matrix can be loaded and processed using R or Python packages such as Seurat^19^ or Scanpy^29^ respectively, followed by further downstream analyses employing tools or software detailed in the methods section.

### 10X Genomics organoid scRNA-seq data

The feature-barcode raw UMI count matrix from 10X Genomics scRNA-seq experiments of the mouse gastro-esophageal organoids is accessible in the GEO repository under the accession code GSE181411^30^. The raw count matrix is available in the MTX format, and the files included are ‘GSE181411_barcodes.tsv.gz’, ‘GSE181411_features.tsv.gz’, and ‘GSE181411_matrix.mtx.gz’. Additionally, the demultiplexed sample data and sample barcode count data are available in CSV format, named ‘GSE181411_demultiplexed_rescued_data.csv.gz’ and ‘GSE181411_raw_MULTISeq_barcount_data.csv.gz’, respectively. All files are provided in a compressed gzip archive format for ease of access and storage.

## Technical Validation

### 10X Genomics scRNA-seq dataset of the gastroesophageal tissue

**Fig. 2:**
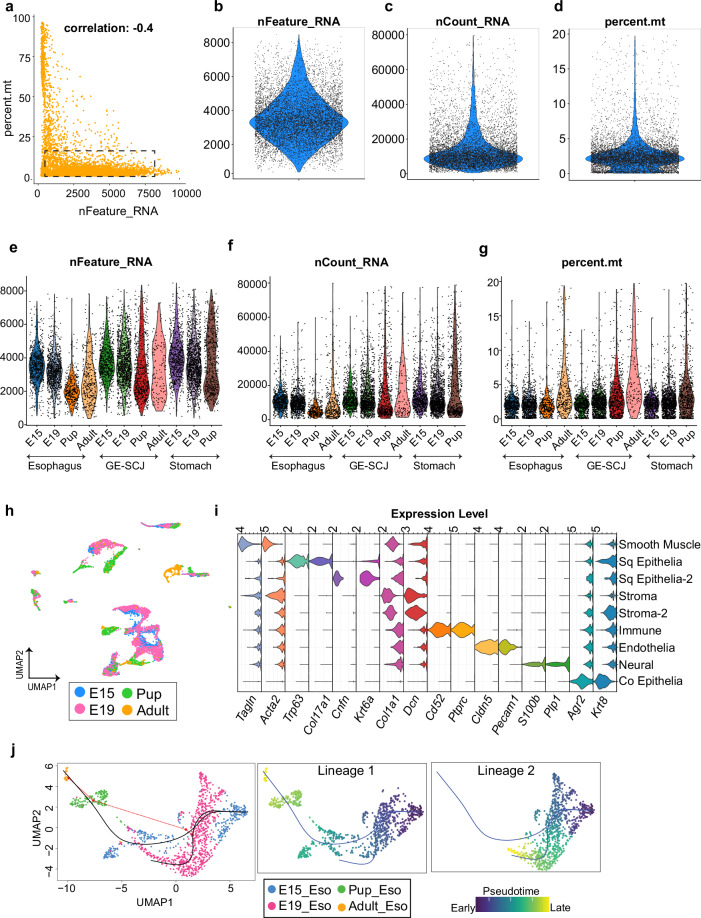
Technical evaluation of scRNA-seq data from E15, E19, pup and adult mice GE-SCJ tissues. **(a)** Scatter plot illustrating the distribution of genes per cell (nFeature_RNA) relative to the percentage of mitochondrial genes (percent. mt). The dotted rectangle emphasizes the region indicating the optimal quality threshold. A Pearson correlation coefficient of −0.4 was observed between nFeature_RNA and percent.mt. **(b**–**d)** Following the quality control (QC) step, violin plots depict the distribution of gene count (nFeature_RNA) (**b**), unique molecular identifier (UMI) count (nCount_RNA) (**c**), and the percentage of mitochondrial genes (percent.mt) (**d**). **(e**–**g)** Violin plots showing the distribution of gene count (nFeature_RNA) (**e**), unique molecular identifier (UMI) count (nCount_RNA) (**f**), and the percentage of mitochondrial genes (percent.mt) (**g**), similar to (**c**–**e**) but at the individual sample level. **(h)** UMAP visualization of all indicated samples showing the cell cluster distribution; cells color-coded by developmental time point. **(i)** Stacked violin plot highlighting the various cell types present in the data along with the expression levels of their respective marker genes. Sq and Co represent squamous and columnar epithelia, respectively. **(j)** UMAP visualization of the pseudotime developmental trajectories observed in esophageal fibroblast cells, originating from the E15 time point; cells arranged according to pseudotime values, progressing from early (dark blue) to late stages (yellow).

The information provided here is related to the evaluation of the 10X Genomics tissue data. Discussions regarding organoid scRNA-seq and the integration of our 10X Genomics data with data derived from other scRNA-seq technologies will be addressed in subsequent sections. The quality of the scRNA-seq libraries was evaluated using several key metrics, including cell statistics, mapping metrics, sequencing metrics, and multiplexing metrics (Table S1). Overall, these metrics demonstrate high library quality and robust data integrity. For example, nearly 90% of reads were confidently mapped to the genome, and Q30 scores for RNA reads, as well for UMIs and barcodes, exceeded 95%. Next, the counts output from CellRanger was imported into R as a Seurat object. Following the standard user guide provided by Seurat, the quality of the imported data was assessed. This involved measuring the number of genes per cell (nFeature_RNA), which allowed for the identification of cell doublets, multiplets (>8500 genes), or empty cells (<100 genes). Additionally, another parameter assessed was the percentage of mitochondrial genes, which serves as an indicator of low-quality cells or dying cells (>20%). However, Fig. 2a illustrates that the majority of cells from our dataset fall within the optimal range, as highlighted by the dotted rectangle. Nonetheless, cells with fewer than 100 genes, more than 8500 genes, and a mitochondrial gene percentage exceeding 20% were filtered out for further analysis, as depicted in Fig. 2b–d. While a 10% cutoff for mitochondrial genes is commonly used for filtering low-quality cells, we opted for a 20% cutoff due to the presence of diverse pre- and post-natal cells from various tissue regions, which may exhibit differing levels of metabolic activity affecting mitochondrial gene expression. In this way, we ensured the retention of biologically meaningful cells while upholding high QC standards, thereby minimizing the risk of discarding potentially informative cells and enhancing the quality of downstream analyses. QC metrics were also visualized at the sample level across tissue regions and developmental stages (Fig. 2e–g). Next, SCT normalization was performed with default settings to remove technical variance. This involved regressing out the effects of cell cycle genes, count depth, and mitochondrial read content.

Dimensionality reduction before clustering was performed on the identified highly variable genes within the dataset. The top 30 principal components were calculated and used as input for K-nearest neighbour graph construction. Subsequently, non-linear dimensionality reduction was performed using the Uniform Manifold Approximation and Projection (UMAP) algorithm. Cell communities were then identified using the Louvain algorithm at a resolution of 0.5 (Fig. 2h). Interestingly, the UMAP in Fig. 2h revealed distinct cell types with unique transcriptional signatures but also delineated cells within these clusters according to developmental time points (evident from the distance between subclusters within the same cell communities). Further analysis utilizing standard canonical markers led to the annotation of these cell clusters, resulting in the identification of 9 different cell types (Fig. 2i). This clearly shows that the dataset preserved cellular heterogeneity of the tissue and captured transcriptional differences within the same cell type at different developmental stages (embryonic to adult). To further explore the progression of developmental stages, we performed pseudotime analysis. Given the complexity of the data, which includes multiple cell types and tissue regions, we focused on fibroblast cells from the esophageal tissue across all developmental time points as a representative example (Fig. 2j). Our analysis revealed two developmental trajectories, both originating from cells at the E15 time point. The first trajectory shows a clear hierarchical transition, with cells from E15 progressing through the E19 subpopulation, then advancing to the pup stage, and ultimately maturing into adult cells. The second trajectory observed indicates a subset of E15 cells differentiating into distinct cell types unique to E19 stage. Collectively, these findings emphasize the dataset’s quality and robustness, indicating its potential for elucidating the developmental progression of any particular cell type of interest. The evaluation of the GE-SCJ tissue data and the rigorous quality control measures used ensure high data integrity, making this dataset a valuable resource for further investigations.

### scRNA-seq data integration and clustering

The integration of our generated 10x Genomics dataset with other scRNA-seq technologies, such as Drop-seq^31^ and CEL-seq2^32^, was conducted using the standard Seurat integration workflow. Prior to integration, scRNA-seq datasets were downloaded from repositories, as described in the Data Records section, and loaded as Seurat objects. The standard SCT normalization workflow was applied according to the user guide, covering all the steps from QC to cell clustering. Due to the variability in cell numbers across the datasets we aimed to integrate, some of which contained a large number of cells, we performed downsampling post-clustering, as described in the methods section.

**Fig. 3:**
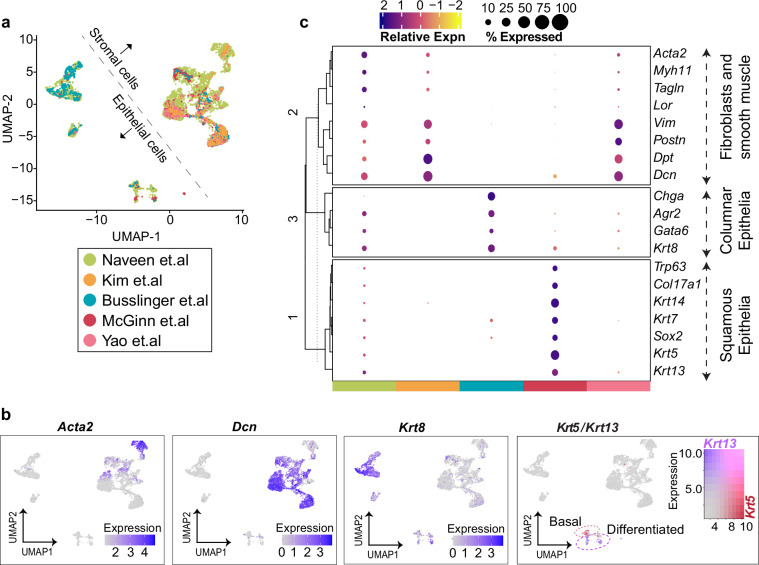
Integration of scRNA-seq datasets reveals consistent cell clustering and gene expression patterns. **(a)** UMAP visualization of epithelial and stromal cell clusters after data integration; cells are color-coded based on their dataset of origin. **(b)** Feature plots showing normalized expression levels of stromal markers *Acta2* and *Dcn*; columnar epithelial marker *Krt8* and squamous epithelial marker *Krt5*/*Krt13*; Basal (red) and differentiated (purple) squamous cells are highlighted by dotted circles. **(c)** Clustered dot plot illustrating the relative expression levels of epithelial and stromal marker genes across the epithelial and stromal cells obtained from their respective scRNA-seq datasets. Each specific cell type from a dataset expresses only markers unique to them. Dot size indicates the percentage of cells expressing a gene, while color represents the scaled mean expression level from high (dark blue) to low (yellow).

Given the presence of diverse cell types and four distinct developmental time points, we opted to focus on assessing the stromal and epithelial cell populations post-integration, as presented here, to reduce complexity. In Fig. 3a, UMAP illustrates a clear separation between stromal and epithelial cell clusters. Importantly, cell clustering remained unaffected by batch effects from different scRNA-seq datasets and was instead driven by cell types and developmental stages, as observed by the well-mixed clustering of cells from different datasets. To confirm the identity of cell clusters, we examined the expression of stromal markers (*Dcn*, *Acta2*) and epithelial markers (*Krt5*, *Krt13*, and *Krt8*), as depicted in Fig. 3b. Within the stromal cell cluster, *Dcn*+ cells representing fibroblasts were distinguishable from *Acta2*+ smooth muscle cells. In the epithelial clusters, columnar epithelial cells expressing the lineage-specific marker, *Krt8* clustered separately, while squamous epithelial cells positive for the lineage-specific marker *Krt5* (basal squamous epithelia) and *Krt13* (differentiated squamous epithelia) were located further apart. Figure 3c highlights the relative expression of marker genes across different scRNA-seq datasets that were integrated. This analysis clearly demonstrates that our dataset contains all epithelial and fibroblast subtypes expressing their associated marker genes. With seamless data integration, cells from external datasets exhibited consistent gene expression patterns post-integration. For instance, stromal cells sourced from Kim *et al*.^22,33^ and Yao *et al*.^25,34^ expressed only stromal cell specific canonical markers. Similarly, columnar and squamous epithelial cells from Busslinger *et al*.^23,35^ and McGin *et al*.^24,36^ datasets, respectively, expressed only their corresponding epithelial lineage-specific markers. This confirms a robust integration without alterations in gene expression patterns.

**Fig. 4:**
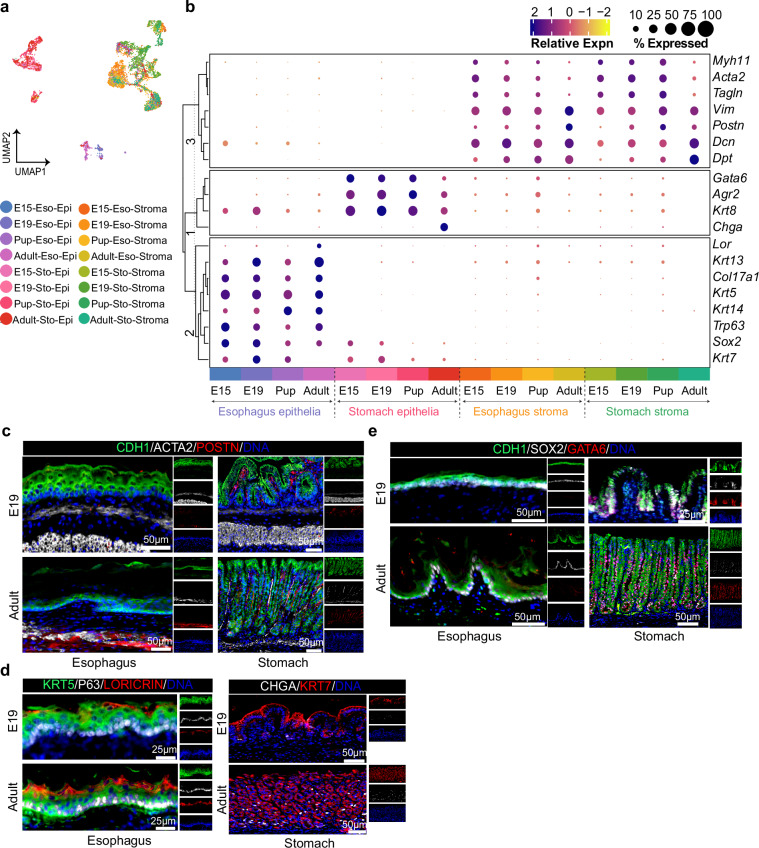
Spatial evaluation of the identified markers from scRNA-seq data. **(a)** UMAP visualization depicting epithelial and stromal cell clusters of esophagus and stomach from various developmental stages; cells are colored according to cell type and time point. **(b)** Clustered dot plot highlighting the relative expression of genes across epithelial and stromal cells of gastroesophageal tissue at developmental stages. Dot size denotes the percentage of cells expressing a gene, while color indicates the scaled mean expression level from high (dark blue) to low (yellow). **(c**–**e)** IHC images of mouse esophagus (left panel) and stomach (right panel) tissue sections at embryonic day 19 (E19, upper panel) and adult stages (lower panel) for various markers. **(c)** CDH1 (green) for epithelia, POSTN (red) for fibroblasts, and ACTA2 (white) for smooth muscle cells. **(d**, left panel**)** KRT5 (green) for squamous epithelia, P63 (white) for basal progenitor cells, and LORICRIN (red) for squamous differentiated cells. **(d**, right panel**)** CHGA (white) for enteroendocrine cells of the stomach base region, and KRT7 (red) for columnar epithelia. **(e)** CDH1 (green) for epithelia, GATA6 (red) for columnar epithelial-specific transcription factor, and SOX2 (white) for squamous epithelial-specific transcription factor, along with nuclei staining (blue). Images represent three biological replicates.

Since the clustering of cells was influenced by both cellular subtypes and developmental time points (Fig. 4a), we assessed the expression of the same set of epithelial and stromal markers at subcluster levels over time (Fig. 4b). This analysis revealed consistent expression patterns corresponding to both cell types and their developmental stages. For instance, genes such as *Krt13* and *Lor*, key markers of terminally differentiated stratified squamous epithelial cells, were highly expressed only during the adult esophagus stage but not at other stages. Similarly, *Chga*, an endocrine cell type marker of the stomach, was expressed highly during the adult phase compared to embryonic stages, indicating the presence of well-developed glandular units comprising distinct cell types in adults. Immunohistochemistry validation in mouse tissue corroborated these inferred cell types and their expression patterns (Fig. 4c–e).

Taken together, these findings underscore the compatibility of our generated data and its robustness in preserving important biological information during integration. Additionally, the results from the integrative analysis of stromal and epithelial cells demonstrate the utility of our dataset for investigating specific cell types of interest, such as immune cells, which play critical roles during tissue injury and repair, infection, and carcinogenic events as well as to explore their evolution and cellular transitions from embryonic to adult stages.

### 10X Genomics scRNA-seq dataset of the gastroesophageal organoids

**Fig. 5:**
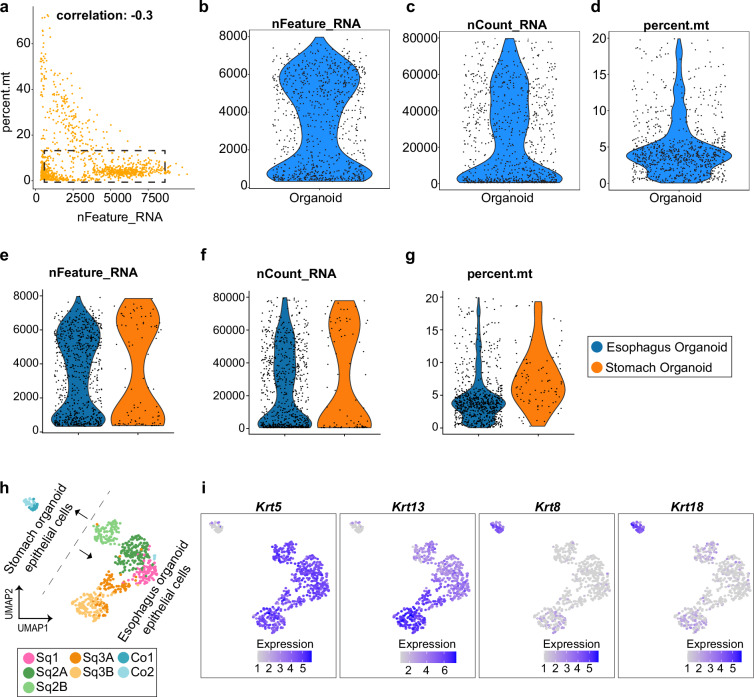
Technical evaluation of the gastroesophageal organoid scRNA-seq data. **(a)** Scatter plot showing the distribution of genes per cell (nFeature_RNA) relative to the percentage of mitochondrial genes (percent. mt). The dotted rectangle indicates the region of optimal quality threshold. A Pearson correlation coefficient of −0.3 was observed between nFeature_RNA and percent.mt. **(b**–**d)** Violin plots depicting the distribution of gene count (nFeature_RNA) (**b**), unique molecular identifier (UMI) count (nCount_RNA) (**c**), and the percentage of mitochondrial genes (percent.mt) (**d**) following the QC step. **(e**–**g)** Violin plots showing the distribution of gene count (nFeature_RNA) (**e**), unique molecular identifier (UMI) count (nCount_RNA) (**f**), and the percentage of mitochondrial genes (percent.mt) (**g**), similar to **(b**–**d)** but at the individual sample level. **(h)** UMAP visualization of gastroesophageal epithelial organoids showing the cellular subtypes; cells color-coded by epithelial cell type. **(i)** Feature plots depicting the absolute expression levels of squamous (*Krt5/Krt13*) and columnar (*Krt8/Krt18*) epithelia; Sq and Co represent squamous and columnar epithelia, respectively.

The processing and analysis of the scRNA-seq data for gastroesophageal organoids followed a similar approach to that used for the GE-SCJ tissue data. Initial evaluation of the gastroesophageal organoid scRNA-seq library demonstrated high quality, for example, with Q30 scores for RNA reads, UMIs, and barcodes exceeding 96%, and nearly 89% of reads confidently mapped to the genome (Table S1). The CellRanger count output from esophagus and stomach organoid was imported into R as a Seurat object. The quality of the acquired data was initially assessed, as shown in Fig. 5a, where we found that the majority of the cells (highlighted by the dotted rectangle) were within the optimal range. However, cells with fewer than 100 genes, more than 8500 genes, and containing mitochondrial reads exceeding 20% were excluded from further analysis, ensuring that only high-quality cells passed this filter (Fig. 5b–d). QC metrics were also visualized at the individual level for both esophagus and stomach organoid samples (Fig. 5e–g).

SCT normalization was performed as described in the Seurat user guide, with default settings to remove technical variance by regressing out the effects of cell cycle and mitochondrial genes. Dimensionality reduction and clustering were conducted using standard functions, similar to those applied to the GE-SCJ tissue dataset. Cell clusters were identified using the Louvain algorithm at a resolution of 1.0 (Fig. 5h). As expected, the UMAP in Fig. 5h clearly shows that the squamous epithelial cells including basal (Sq1), parabasal (Sq2), and terminally differentiated (Sq3) from esophageal organoids clustered separately from the columnar epithelial cells comprising those from the base, neck, and isthmus region (Co1), as well as the pit region (Co2) of the stomach organoids. Gene expression levels of the selected markers in Fig. 5i clearly highlight the transcriptional differences between the two epithelial lineages. Thus, this dataset complements the tissue scRNA-seq data and serves as a valuable asset to validate gene signatures and regulatory factors, as it demonstrates two key aspects: (i) stem cell-derived epithelial organoids from the gastroesophagus can recapitulate the *in vivo* cellular architecture; and (ii) the generated dataset is robust in capturing cellular heterogeneity and their transcriptional signatures.

## Usage Notes

### Potential limitations of the datasets

Overall, the data generated from different time points across the gastroesophageal tissue regions were of high quality, indicating the robustness of our approach. While encountering an issue where no cells of the adult stomach being assigned with a CMO barcode, we acknowledge potential challenges during cell handling, library preparation, or sequencing. Consequently, this sample was excluded from data analysis. However, despite these challenges, the integration of four external scRNA-seq datasets compensated for the data gap and facilitated spatial validation of identified cell types, highlighting the complementary nature of our generated data pre- and post-integration. Nevertheless, the datasets generated here offer a wealth of information on the transcriptional regulation and evolutionary dynamics of the GE-SCJ regions from embryonic to adult mice. To the best of our knowledge, this study represents the first time-series transcriptomic analysis of the esophagus, stomach and GE-SCJ regions within the upper gastrointestinal tract. We firmly believe that the generated data hold considerable value in the field of cell and developmental biology, offering new avenues for investigating mechanisms driving disorders and malignancies of the gastroesophagus.

## Supplementary information

Supplementary Table S1

## Data Availability

scRNA-seq raw data processing and sample demultiplexing was performed using the Cell Ranger software (downloaded from 10x genomics official website). Organoid scRNA-seq sample demultiplexing was performed using deMULTIplex (https://github.com/chris-mcginnis-ucsf/MULTI-seq). scRNA-seq data analysis and integration was conducted using Seurat (https://satijalab.org/seurat/). No custom code was generated in this study.
